# Post-metamorphic skeletal growth in the sea urchin *Paracentrotus lividus* and implications for body plan evolution

**DOI:** 10.1186/s13227-021-00174-1

**Published:** 2021-03-16

**Authors:** Jeffrey R. Thompson, Periklis Paganos, Giovanna Benvenuto, Maria Ina Arnone, Paola Oliveri

**Affiliations:** 1 Department of Genetics, Evolution and Environment, University College London, Darwin Building, Gower Street, London, WC1E 6BT UK; 2 UCL Center for Life’s Origins and Evolution, London, UK; 3 Stazione Zoologica Anton Dohrn, Villa Comunale, 80121 Naples, Italy

**Keywords:** Echinoid, Skeleton, Development

## Abstract

**Background:**

Understanding the molecular and cellular processes that underpin animal development are crucial for understanding the diversity of body plans found on the planet today. Because of their abundance in the fossil record, and tractability as a model system in the lab, skeletons provide an ideal experimental model to understand the origins of animal diversity. We herein use molecular and cellular markers to understand the growth and development of the juvenile sea urchin (echinoid) skeleton.

**Results:**

We developed a detailed staging scheme based off of the first ~ 4 weeks of post-metamorphic life of the regular echinoid *Paracentrotus lividus*. We paired this scheme with immunohistochemical staining for neuronal, muscular, and skeletal tissues, and fluorescent assays of skeletal growth and cell proliferation to understand the molecular and cellular mechanisms underlying skeletal growth and development of the sea urchin body plan.

**Conclusions:**

Our experiments highlight the role of skeletogenic proteins in accretionary skeletal growth and cell proliferation in the addition of new metameric tissues. Furthermore, this work provides a framework for understanding the developmental evolution of sea urchin body plans on macroevolutionary timescales.

**Supplementary Information:**

The online version contains supplementary material available at 10.1186/s13227-021-00174-1.

## Background

The evolution of animal body plans has resulted in the vast diversity of animal morphologies seen in deep time and on the planet today [1]. Crucial for understanding the evolution of animal morphology is, however, a precise understanding of the molecular and cellular mechanisms which operate during animal growth and development [2]. Skeletons, the hard, biomineralized tissues which provide structure and support for numerous animals, make up the majority of the animal fossil record, but are also ideal systems for understanding the genetic and cellular changes that take place during body plan development and evolution [3]. Echinoderms, the clade of deuterostomes including starfish and sea urchins, have a biomineralized endoskeleton made up of a porous meshwork of CaCO_3_ and comprising numerous interlocking and abutting skeletal plates [4]. The echinoderm skeleton has conferred upon the group an exceptional fossil record, precisely demonstrating evolutionary changes in body plans [5, 6]. Within the echinoderms, the sea urchin (echinoid) larval skeleton has also become a paradigm for understanding the mechanistic basis for development, and the genetic regulatory networks operating in larval echinoid skeletal development are exceptionally well-understood [7, 8]. While there is a breadth of knowledge concerning the development and evolution of the larval skeleton in sea urchins, there remains a poorer understanding of the development of the juvenile sea urchin skeleton at the level of the gene, protein, and cell.

**Fig. 1:**
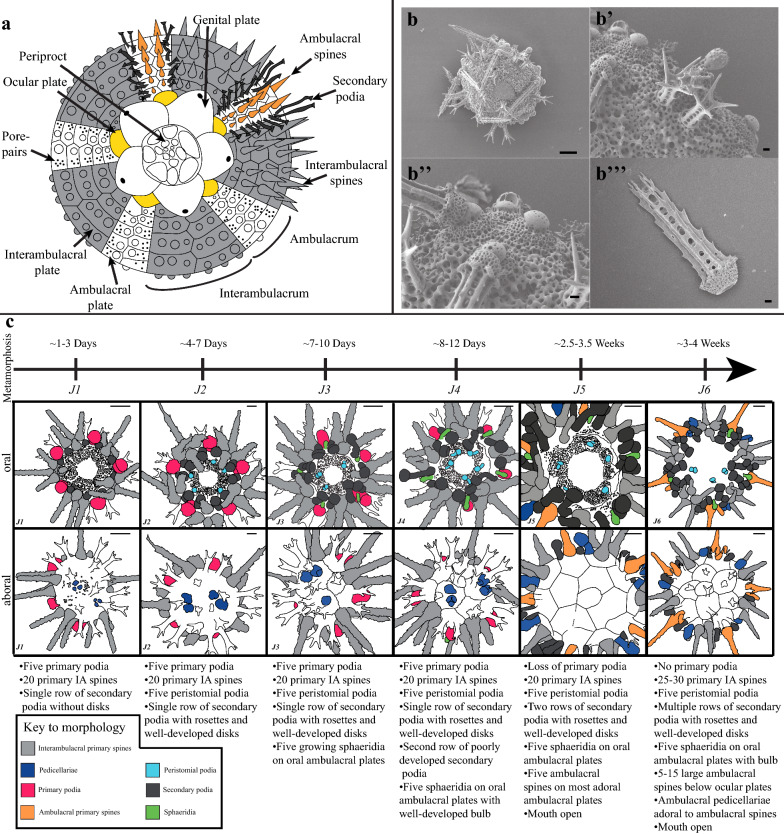
**a** Diagram modified from [46] showing morphology of adult sea urchin test from adoral view. **b** SEM image showing aboral view of test with juvenile and primary spines Scale bar is 100 μm. **b’** Close-up of b showing juvenile spines, ambulacral spine, and sphaeridia. **b**’’ Close-up of b showing the details of the interambulacra. Primary interambulacral spines articulate with the bosses of primary tubercles on interambulacral plates. **b’’** Isolated primary spine. Scale bars in (**b’**–**b’’**) 10 μm. **c** Staging scheme showing example drawings and descriptions of each stage and the addition of morphological structures through the first 4 weeks of development. Drawings in **c** are based off of traces of fixed and stained individuals. Structures are color coded as in the figure legend. IA, interambulacral. Scale bars in **c** are 100 μm

Most echinoids, like many marine invertebrates, have a biphasic life style. Following a protracted larval stage, the adult or juvenile body plan emerges from the larvae during metamorphosis [9]. The post-metamorphic sea urchin body plan is pentaradially symmetrical, globe shaped, and comprised multiple CaCO_3_ tessellate plates which make up the test (Fig. 1). Depending upon the species, test plates are covered in one or multiple protrusions called tubercles, which attach to spines via a ball-and-socket joint (Fig. 1). The skeletal tissues of the echinoid test have been historically demarcated into axial and extraxial structures [10]. Axial structures include the ocular and ambulacral plates, skeletal plates through which the tube feet of the water vascular system protrude, and the interambulacral plates, which, in juveniles, are characterized by large primary spines (Fig. 1a, b). New axial skeletal elements are added to the test from a growth zone at the margin of the ocular plates (Fig. 1a) [11]. Extraxial elements of the echinoid test are confined to the most aboral (farthest from the mouth) skeletal elements of the test, and include the periproctal and genital plates (Fig. 1a). Extraxial elements contrast with axial elements because they are not added via a distinct growth zone.

Growth of the echinoid test has been attributed to two distinct processes, plate addition and plate accretion [12]. Plate addition is characterized by the formation of new test plates at the margin of the ocular plates [11]. As new plates are added, and the animal grows larger, the position of these plates relative to the ocular plate shifts closer to the oral surface, the surface bearing the mouth. This process is tightly linked to the addition of other metameric structures of the echinoid test such as the secondary podia [13–15]. Plate accretion is the accumulation of new skeletal material onto the margins of already-added plates. This process results in the elaboration of test plate morphology, and is reminiscent of growth and remodeling of vertebrate bone [16].

Despite the long history of work characterizing changes in echinoid morphology through ontogeny [9, 11, 12, 17–21], there has been relatively little work tying these morphological changes to their molecular and cellular underpinning. To fill this gap, we performed detailed analyses of morphological changes during growth, immunohistochemistry, and fluorescent assays of skeletal growth and cell proliferation in post-metamorphic juveniles of the sea urchin *Paracentrotus lividus*. This has shed light on the molecular and cellular processes operating during juvenile sea urchin growth, with a focus on the processes of skeletal plate addition and accretion.

## Results

### Stages of juvenile growth

During growth of the late larvae, the adult body plan develops (Additional file 1: Fig. S1) and the pentaradial juvenile emerges during metamorphosis [9]. After metamorphosis, juveniles are approximately 200–400 μm in diameter and have distinct ambulacral and interambulacral areas with spines and primary podia. Juveniles grow rapidly following metamorphosis, adding additional structures such as spine-like sensory sphaeridia, which may be involved in environment sensing and balance [22], and pincer-like pedicellariae (Fig. 1b, c). Post-metamorphic growth is asynchronous across and within cultures. Thus to make standardized comparisons across different individuals, morphological characteristics of ~ 100 animals from two cultures were analyzed daily from 0 to 4 weeks post-metamorphosis and grouped based on morphological differences. This resulted in a staging scheme based on the presence or absence of morphological features encompassing six distinct stages herein termed J1–J6 (Fig. 1c; Additional file 1: Fig. S2). Stages are summarized in Fig. 1, and a detailed description is given in the Additional files 1: Data.

### Molecular characterization of juvenile tissues

**Fig. 2:**
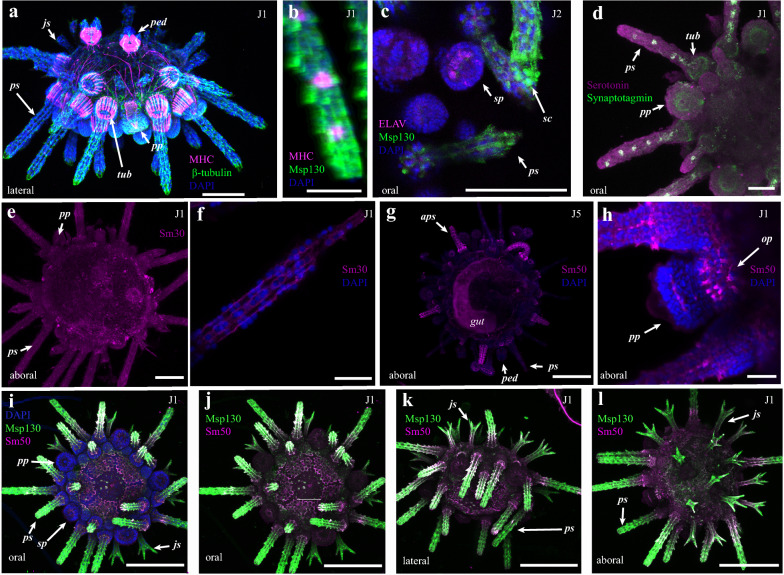
Molecular characterization of juvenile *P. lividus* cell and tissue types. a Staining with antibodies against Msp130 and β-tubulin reveals the distribution of musculature. Details in text. **b** Staining against Msp130 and MHC shows MHC^+^ cells within the spines. **c** Staining against ELAV and Msp130 shows nerves in the secondary podia, and skeletogenic cells in the spines. **d** Staining for synaptotagmin and serotonin reveals the extent of the nervous system and serotonergic neurons. **e**–**h** Immunostaining using antibodies against the skeletogenic proteins Sm30 and Sm50 stains skeletal tissues, with stronger staining in more recently deposited biomineral. **i**–**l** Localization of the skeletogenic proteins Msp130 (green) and Sm50 (purple) in a J1 *P. lividus* juvenile. Description of stainings given in the main text. js, juvenile spine; ps primary interambulacral spine; ped, pedicellariae; aps, ambulacral primary spine; pp, primary podia; sp, secondary podia; tub, tubercle; gut, gut; op, ocular plate, gp, genital plate; ap, anal plate; sph, sphaeridia; al, Aristotle’s lantern. Scale bars in **a**, **c**, **e** are 100 μm; **d** is 50 μm; **b**, **f**, **h** are 25 μm, and **g**, and **i**–**l** are 200 μm

In order to understand the molecular basis for the development of the structures identified in our staging scheme, immunohistochemical markers were used to characterize tissue and cell types in *P. lividus* juveniles (Fig. 2a–l). To visualize muscular tissues, an anti-myosin heavy chain (MHC) antibody that identifies and localizes muscles surrounding the gut of larval echinoderms [23] was used. Staining with the anti-MHC antibody (*n* = 7) shows reactivity along the interior of the tube feet (Fig. 2a, Additional file 1: Figs. S3, S4), in longitudinal bands surrounding the base of spines and tubercles (Fig. 2a, Additional file 1: Fig. S3a, S4a–d), in bundles along the length of the spines (Fig. 2b, Additional file 1: Fig. S3b, S4d), in muscles supporting the jaws or Aristotle’s lantern and, most strongly, in the base of the pedicellariae (Fig. 2a, Additional file 1: Figs. S3c, S4c). This is comparable to immunoreactivity recently shown for F-actin [24].

To identify and characterize the structure of the juvenile nervous system, samples were incubated with antibodies against pan- and specific neuronal markers (Fig. 2c–d). These results are largely in agreement with a recent characterization of the *P. lividus* nervous system [24]. 1E11, a pan-neuronal marker of the synaptic vesicle trafficking protein synaptotagmin [25–27], was used to identify the distribution of neurons, neuronal projections, and synapses (Fig. 2d, Additional file 1: Fig. S5). 1E11 identified bundles of fibers that form a ring around the bases of the tubercles, in the tube feet, and in cells along the lumen of the spines (*n* = 5; Fig. 2d). In contrast to the pan-neuronal synaptotagmin, an antibody against serotonin identified a smaller subpopulation of serotonergic neurons along the lumen of the interambulacral primary spines where they co-localize with synaptotagmin. (*n* = 1; Fig. 2d, Additional file 1: Fig. S5). Another neuronal marker is ELAV, an RNA-binding protein used to identify neurons [25]. Isolated ELAV^+^ cells were found in the primary and secondary podia (*n* = 1; Fig. 2c, Additional file 1: Fig. S6) in a pattern reminiscent of sensory motor neurons found in podia of ophiuroids [28]. Importantly, these cells are distinct from the skeletal tissues of the rosette (Fig. 2c, Additional file 1: Fig. S6b and Sd). Co-staining with anti-ELAV and 1E11 revealed co-reactivity in cells in the primary interambulacral spines (Additional file 1: Fig. S7). Acetylated and β-tubulin have also been used to characterize neurons in other animal groups. An anti-acetylated tubulin antibody reacted with numerous cilia covering the surface of J1 individuals, and distributed in a circular pattern around the margins of the distal ends of podia. Staining is also present along the interior of newly forming secondary podia, which may be the neural plexus of [24] (*n* = 2; Additional file 1: Fig. S8). An anti-β-tubulin antibody showed similar results (Fig. 2a, Additional file 1: Fig. 4c, f) in marking tissues of the nervous system, and cilia on the surface of J1 and J2 animals (*n* = 6).

Lastly, to visualize developing skeletal tissues and skeletogenic cells, antibodies were used against three well-characterized proteins known to be involved in echinoid skeletal growth and development. The first of these are the spicule matrix protein 50 kDa (Sm50) and the spicule matrix protein 30 kDa (Sm30) [29], two c-lectin type extracellular matrix proteins occluded within the biomineral of the larval and adult skeleton [30–33]. Staining at different stages with both Sm30 and Sm50 show immunoreactivity with all skeletal structures except for the juvenile jaws and teeth (*n* = 11; Fig. 2e–l). Crucially, and consistent with differential staining in the larvae and growing juvenile skeleton (Additional file 1: Figs. S1, S9), staining is stronger in newly formed skeletal elements (identified based on our staging scheme) such as growing ocular plates in J1 individuals and newly added ambulacral spines in J5 animals (Fig. 2g–h). Another marker for skeletal cells is the cell-surface glycoprotein Msp130, known to be specifically expressed in skeletogenic cells and skeletal tissues of larval and adult sea urchins [30–32]. The monoclonal antibody 6a9 reacts specifically with Msp130 [34]. In different stages in juvenile *P. lividus,* immunostaining with 6a9 identifies all skeletal tissues except for the jaws and teeth (*n* = 10; Fig. 2i-l, Additional file 1: Fig. S9), and in some cases clearly marks cell bodies of skeletogenic cells (as in [24]), which can be seen in the interambulacral spines (Fig. 2c, Additional file 1: Fig. S6). Application of these antibodies in juvenile *P. lividus* has led to a more detailed understanding of the molecular signatures for their musculature, nervous system, and skeleton. In particular, antibodies against skeletal proteins facilitate further study of skeletal formation and growth in post-metamorphic sea urchins.

### Growth and skeletogenesis of plate accretion

During juvenile growth, skeletal elements like plates and spines are constantly remodeled and elaborated upon. Plate accretion is the process by which new skeletal tissue is added onto pre-existing skeletal structures [12]. During larval development, cells in the growing portions of the larval skeleton, such as the tips of the arms, express distinct sets of genes (i.e., *Sm30*, *Sm50* and *Msp130*) relative to other skeletogenic cells [35, 36]. We thus hypothesized that plate accretion might similarly involve a distinct set of proteins. To understand the extent to which proteins are differentially or distinctly localized during skeletal growth, and precisely visualize plate accretion, double-fluorescent immunostaining of skeletal markers (6a9 specific to Msp130, and anti-Sm50) was combined with fluorescent labeling of the growing CaCO_3_ skeleton. In J1 J3 and J6 *P. lividus,* both antibodies are co-localized in all skeletal structures except for the teeth (*n* = 7; Fig. 2i–l, Additional file 1: Fig. S9a–f). Stronger immunoreactivity for both antibodies was identified in newly formed skeletal tissues, such as the margins of peristomial, ambulacral and interambulacral plates (Fig. 2i–l). The strength of staining of antibodies varies in a tissue-specific manner (Fig. 2i–l), with Msp130 distinctly localized in more distal portions of primary and juvenile spines (Fig. 2i–l, Additional file 1: Fig. S9) and Sm50 more strongly localized in the edges of growing coronal and peristomial plates, in the rosettes of primary and secondary podia, and the bases of newly formed spines in J6 animals (Fig. 2g, i–l). In addition to localizing differentially, Sm50 and Msp130 antibodies strongly co-react in the median portions of primary spines (Fig. 2i–l), and co-react less strongly throughout almost all other skeletal tissue. This suggests that these proteins likely co-localize during post-metamorphic skeletal growth.

**Fig. 3:**
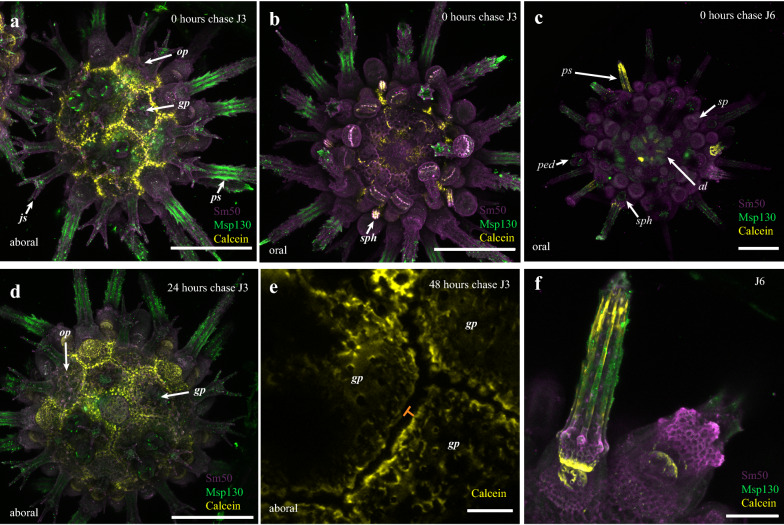
Skeletogenesis in juvenile *P. lividus*. **a** Localization of Sm50 (purple), Msp130 (green) and incorporation of calcein (yellow) in aboral surface of J3 individual at 0 h chase. **b** Oral surface of **a**. **c** Sm50, Msp130 and calcein in J6 animal at 0 h chase. **d** Aboral surface of 24 h chase J3 individual. **e** Zoom of calcein incorporation in margins of aboral plates in 48 h chase J3 individual. Orange bars indicate gap between calcein-marked skeleton due to subsequent accretion. **f** Close-up of the aboral surface showing incorporation of calcein into growing spines and tubercle. Abbreviations as in Fig. 2 and scale bars in **a**–**d** are 200 μm, **e** is 25 μm, and **f** is 50 μm

In addition to immunostaining, calcein [35] was used to identify sites of active skeletogenesis and their distribution relative to the localization of skeletal proteins (Fig. 3a–f). Calcein is a fluorescent marker that binds to Ca^2+^ and is incorporated into the CaCO_3_ skeleton during biomineral deposition [35]. Calcein staining is visible in the sea urchin skeleton following fixation and immunostaining and discrete pulses of incubation with calcein label only skeletal structures growing during the period of incubation. Pulse-chase experiments can thus be used to not only to visualize the position of calcite deposition, but also to determine the spatial extent of subsequent skeletal growth following calcein incubation. J3 (*n* = 5) and J6 (*n* = 1) animals were incubated with calcein for a period of 24 h, then calcein was washed out and replaced with SW. Animals were fixed and observed immediately after calcein staining (0 h), or 24 and 48 h after removal. Figure 3 shows the results of calcein staining in J3 and J6 *P. lividus* juveniles, respectively.

After 24 h of incubation (0 h chase) in J3 animals, calcein was incorporated into the margins of skeletal structures and plates on both the oral and aboral surfaces. Ocular plates, genital plates, and the anal plate all show calcein incorporated into their growing periphery (Fig. 3a, d, e, Additional file 1: Figs S10–S12). Calcein was also incorporated into the growing margins of interambulacral, ambulacral, and peristomial plates, as well as in elongating sphaeridia and in the rosettes of growing secondary podia (Fig. 3a–b, Additional file 1: Figs. S10–S12). On the oral surface, growing hemipyramids of the Aristotle’s lantern (the masticatory apparatus) also show strong incorporation of calcein (Fig. 3b). In the J6 animal, calcein was incorporated into newly forming ambulacral and interambulacral spines, their corresponding tubercles, as well as in the rosette of a newly added secondary podium (Fig. 3c, f, Additional file 1: Fig. S13a–d). In J3 animals, the most striking calcein-labeled structures are the growing hemipyramids (Fig. 3b), while in J6 animals they are the growing teeth (Fig. 3c, Additional file 1: Fig. S13a–b). This indicates that substantial skeletogenesis is not only taking place in the plates of the test, but also the Aristotle’s lantern prior to the opening of the mouth [24]. Lack of calcein in spines of J3 animals (Fig. 3a, b, d), but presence in plates, and relative lack of calcein in J6 plates later in development (Fig. 3c, f) suggests modular, piecewise growth. This implies distinct skeletal units grow at distinct times.

In 0 h chase animals, calcein incorporated into the margins of plates abuts with calcein incorporation in adjacent plates (Fig. 3a, Additional file 1: Fig. S11, S12). In 24 and 48 h chase animals, however, there are gaps between the calcein-marked skeleton in adjacent plates, indicating further growth post-incubation (Fig. 3a, d, e). Proximal to distal skeletal growth is also visible in the sphaeridia of 24 and 48 h chase animals, where calcein is incorporated into the bases of these structure, but not growing tips (Additional file 1: Figs. S11, S12). Newly synthesized skeletal structures marked by calcein, and skeleton deposited post-calcein incubation, show strong immunoreactivity with Msp130 (6a9) and Sm50 antibodies (Fig. 3, Additional file 1: Fig S10, S11). This can be seen clearly on the aboral surfaces of the animals in Fig. 3a, b (Additional file 1: Fig. S12a–c). This further supports the interpretation that skeletogenic proteins are more abundant in sites of skeletal growth, as is the case in sea urchin larvae [35, 36]. Taken together, these data clarify the morphological changes associated with skeletal growth via accretion and implicate the involvement of skeletogenic matrix proteins. Furthermore, as in the larvae, localization of these proteins suggests that they have both shared and distinct uses in development of the post-metamorphic sea urchin skeleton.

### Cell proliferation in juvenile growth

**Fig. 4:**
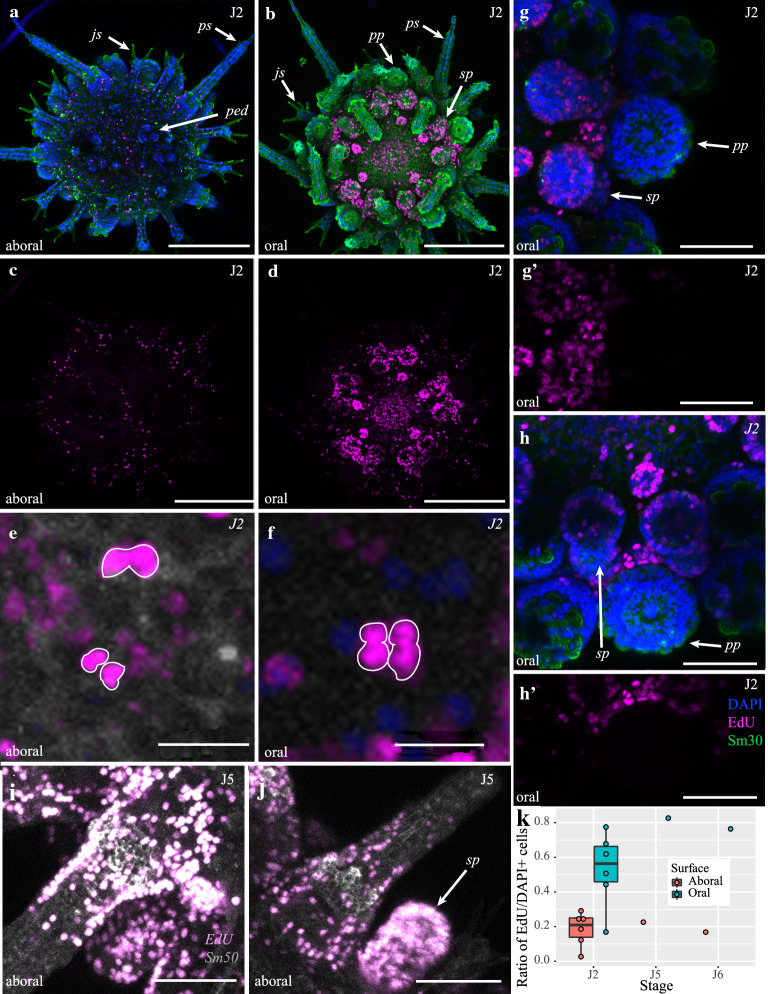
Growth via cellular proliferation in*. P. lividus*. **a**–**h**’ Proliferation on aboral (**a**, **c**) and oral (**b**, **d**, **e**, **f**, **g**–**h**’) surfaces in J2 animals. **e**, **f** Close-ups of proliferating cell doublets in 24-h post-chase animal (**e**) and quadruplets in 48 h post-chase animal (**f**). **g**–**h**’ Zoom showing proliferative zone associated with plate addition. **i**–**j** Cell proliferation associated with the addition of new ambulacral spines and secondary podia in J5 animal. **k** Graph showing differential cell proliferation on oral and aboral surfaces. Abbreviations as in Fig. 2. Scale bars in **a**–**d** 200 μm, **e**–**f** 10 μm, **i**–**j** 100 μm, and **g**–**h** 50 μm

So far, we have shown that juvenile sea urchin test growth relies on extensive elaboration of pre-existing skeletal structures. Our staging scheme also highlights the addition of new skeletal plates and associated structures such as spines, podia, and pedicellariae during growth. Therefore, it is plausible that cell proliferation might underlie the addition of new morphological structures during the course of echinoid post-metamorphic growth. To identify and quantify proliferating cells during juvenile growth, we used 5-ethynyl-2′-deoxyuridine (EdU), a nucleoside analogue of thymidine incorporated into newly synthesized DNA, to label the nuclei of dividing cells. We then used confocal microscopy to image and quantify EdU^+^ cells on both the oral and aboral surfaces of the test of eights individuals from stages J2, J5 and J6 (Fig. 4a–k, Additional file 1: Figs. S14–S17). Overwhelmingly, more EdU^+^ cells were present on the oral surface than the aboral surface (Fig. 4a–h, k). To make sure this was not an artifact due to differences in cell densities on each surface, we calculated the ratio of EdU^+^/DAPI-stained nuclei as described in Additional file 1: Methods (Additional file 1: Fig. S14, Additional file 2, Additional file 3: Table S2). Data clearly show significantly higher ratios of proliferating to non-proliferating cells on the oral surface than the aboral surface (Fig. 4a–h,k; Mann–Whitney *U* test, *p* = 0.0014, *U* = 59) a pattern also evident in raw cell counts (Additional file 3: Table S2).

On the oral surface, EdU^+^ cells are concentrated in growing secondary and peristomial podia (Fig. 4g, h). This contrasts with the primary podia, which form as coelomic outgrowths prior to metamorphosis ([21]; Additional file 1: Fig S1), and display no evidence of cell proliferation (Fig. 4g–h, Additional file 1: Fig. S15). Immediately towards the oral surface of the primary podia and ocular plate, there is a distinct zone of cell proliferation (Fig. 4g–h). The location of the proliferative zone is crucial, as it corresponds to the position where new plates form during plate addition, and where new secondary podia grow [37]. Pulse-chase experiments (Additional file 1: Fig. S15) show similar results between 0 and 48 h chase, though the presence of nuclei doublets and rare quadruplets in 24 and 48 h chase individuals indicate relatively slow rates of cell division (Fig. 4e–f, Additional file 1: Fig. S15). Assays of cell proliferation were paired with antibodies against Sm30 and Sm50, to identify where cell proliferation takes place relative to skeletal growth. There is a general correspondence of EdU^+^ cells to newly grown or growing skeletal structures, such as ambulacral spines in J5 individuals (Additional file 1: Fig. S16f). There are few EdU^+^ cells at the distal ends of these structures. In J6 individuals, other growing structures, such as new pedicellariae and secondary podia, are associated with an abundance of EdU^+^ cells (Additional file 1: Fig. S17). This indicates that addition of new morphological structures relies on cell proliferation. The absence of proliferating cells at the distal ends of spines, however, suggests that elaboration and further growth these structures rely less on proliferation, and more on biomineral deposition via differentiated cells, in agreement with our calcein staining (Fig. 3f). Assays using EdU thus show that the majority of growth via cell proliferation takes place on the oral surface, while structures on the aboral surface are much less proliferative. Furthermore, immediately below the primary podia there is a high-density of proliferative cells in correspondence with the location of plate addition, suggesting cell proliferation underlies the addition of new test plates of the echinoid test. In addition to new test plates, the growth of new pedicellariae, spines, and tube feet is also associated with high degrees of cell proliferation.

## Discussion and conclusions

### Adult growth in echinoderms

The growth of juvenile *P. lividus* shows similarities to the growth of other echinoderms, and our experiments shed light on the molecular, cellular, and morphological underpinning of echinoderm growth generally. All eleutherozoans continue to grow throughout their lifetime by adding new axial skeletal elements from a growth zone [10]. While in asterozoans this zone of growth is adjacent to the terminal podium, the most distal position of the arm, in echinoids it is directly abutting the five ocular plates [10]. It is of interest then, that in just-metamorphosed juveniles of *P. lividus,* the location of the growth zone is located similarly to its position in flattened early post-metamorphic asterozoans. This highlights the fact that the morphology of eleutherozoans begins to diverge extensively after metamorphosis rather than during rudiment development.

Immediately below the ocular plate, our analyses identified a region of proliferating cells coinciding with the zone of plate addition. This zone has been hypothesized as a signaling center responsible for the addition both columns of ambulacral plates, and flanking columns of interambulacral plates, in each ray [10] (Fig. 1). Our assays provide insight into the mechanism by which plates are added; namely that the origin of axial tissues formed in each of these paired regions is associated with the proliferation of new cells. Recent knockouts of the pigment genes *Pks* and *Gcm* in the regular echinoid *Hemicentrotus pulcherrimus* support the hypothesis that cell lineages in the echinoid test are partitioned into ten paired regions, each consisting of a half-ambulacrum and half-interambulacrum [38]. Paired with our data on cell proliferation, we hypothesize the existence of 10 discrete aboral proliferative zones, corresponding with each of these paired regions.

### Molecular and cellular underpinning of skeletal growth

**Fig. 5:**
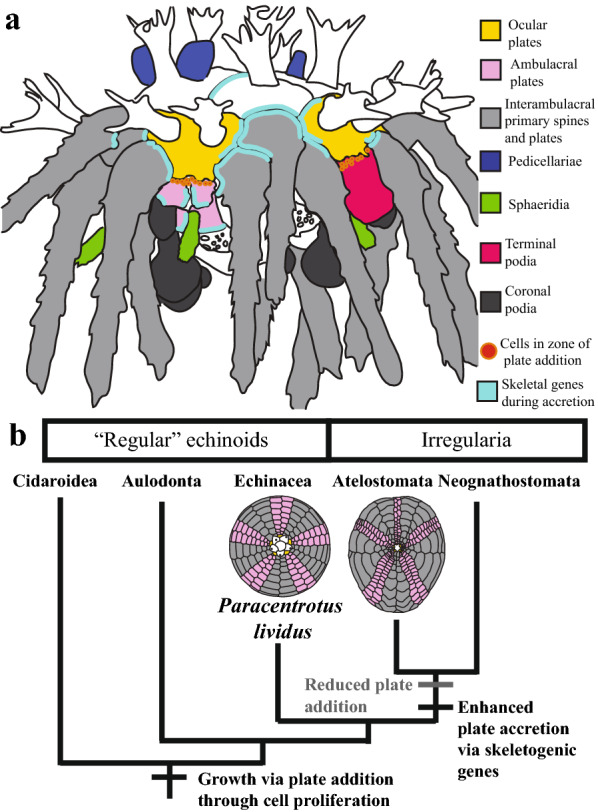
**a** Summary diagram of cell proliferation and expression of skeletal genes during plate accretion and addition in juvenile test growth. **b** Simplified phylogenetic tree of crown group echinoids showing the transitions in sea urchin growth modes in regular and irregular echinoids, and their hypothesized molecular and cellular foundations. Tree is based on [43] and [47]

Our results suggest that both plate addition and plate accretion contribute to the growth of the sea urchin adult body plan and rely on different molecular and cellular processes (Fig. 5a). Growth at the margins of pre-existing test plates is associated with the intense localization of skeletogenic proteins such as Msp130 and Sm50 (Fig. 5a). This is consistent with proteomic analyses that have identified proteins of the Msp130 and Sm families in the organic matrix of tests, spines and teeth of adult sea urchins [30, 31]. A previous study using whole mount in situ hybridization found the skeletogenic gene *Sm37*, the transcription factor *Alx1*, and the signaling molecule *VegfR* expressed in the margins of test plates of juvenile echinoids [39]. In the skeletal cells of embryonic and larval sea urchins, *Alx1* and *VegfR* regulate the expression of skeletogenic genes like *Sm30, Msp130* and *Sm50* [8]. The precise localization of the skeletogenic proteins Msp130 and Sm50 relative to growing skeletal structures in our calcein experiments suggests that upstream regulators such as *Alx1* and *VegfR*, are also important for plate accretion (Fig. 5a).

The addition of new test plates and their associated water vascular and nervous system tissue has been suggested to be analogous to a posterior growth zone as in vertebrates and arthropods [13, 15, 20, 37, 40, 41]. Our results support the idea that the addition of new metameric skeletal structures is associated with cell proliferation. The proliferative zone directly below primary podia is located in the site of plate addition, suggesting the addition of new test plates from this region is associated with cell proliferation. We can speculate that this proliferative zone may use mechanisms involved in metameric growth across the animal kingdom. Comparative studies have identified Wnt signaling and posterior Hox gene expression as evolutionarily conserved players in posterior growth [42], and such a zone has been hypothesized based on the expression of *Hox11/13* temporally earlier in development of the adult echinoid body plan [13, 15]. Outside of this growth zone, high degrees of cell proliferation in newly added ambulacral spines and pedicellariae indicate that proliferation of cells is also involved in the development of skeletal structures. Indeed, the presence of proliferating cells surrounding structures with strong staining of Sm50 suggests that the growth of new anatomical structures may rely on cell proliferation, which may precede skeletal accretion via differentiated cells. Similarly, during regeneration in ophiuroids, differentiated skeletogenic cells which secrete the skeletal plates of the arms are not proliferative [41]. The absence of proliferating cells at the distal tips of newly skeletonizing spines in *P. lividus* suggests a similar scenario.

### Accretion, addition and body plan evolution

Extant echinoids are classified based upon the organization of their body plans into the globe-shaped regular echinoids, which include *P. lividus*, and the irregular echinoids or Irregularia, a clade of bilaterally symmetrical, flattened and oblong forms including sand dollars, heart urchins, and sea biscuits (Fig. 5b) [43]. Regular echinoids are not a clade, but are paraphyletic with respect to the Irregularia, and the fossil record provides a precise window into the morphological transitions that characterize the evolution of irregular echinoids from their regular echinoid ancestors [44]. Extant regular echinoids have displayed marked morphological constraint in the ~ 270 million years since their ancestors were alive. In contrast, the irregular echinoids have drastically diversified their body plans relative to their regular echinoid ancestors, displaying high phenotypic disparity and heightened rates of morphological evolution [5, 6]. It has been hypothesized that the differential diversity of regular and irregular echinoids is due to differential reliance on the processes of plate addition and accretion during test growth [12]. Regular echinoids rely primarily on continuous new plate addition throughout their lives, while irregular echinoids cease adding plates early in their post-metamorphic ontogeny, and instead grow primarily by accreting onto pre-existing plates [12, 17]. Our experimental results from *P. lividus* allow us to make testable hypotheses concerning the role of cellular processes and gene expression in the evolutionary transition from regular to irregular echinoid body plans. Because irregular echinoids rely less on plate addition, and more on plate accretion, we expect that they grow less via proliferation of new cells. Instead, we hypothesize that they rely more on the activity and expression of skeletogenic genes operating during enhanced plate accretion such as *Sm50* and *Msp130,* to grow their tests (Fig. 5b). In light of our results and given their enhanced reliance on biomineral deposition, we hypothesize that irregular echinoids may have diversified the skeletogenetic toolkit via instances of duplication and/or subfunctionalization of skeletogenic genes throughout their evolutionary history. Future analyses of emerging transcriptomic and genomic datasets will shed light on this hypothesis.

## Methods

### Culturing and staging scheme

Two cultures of larvae from different pairs of adult *P. lividus* were reared through to metamorphosis at the culturing facilities at the Stazione Zoologica Anton Dohrn (Additional file 1: Methods). After metamorphosis, which took place over the period of about two to three days, approximately 50 post-metamorphic juveniles were present in each culture. As cultures developed, animals were examined daily using a ZEISS Stemi 2000 stereomicroscope, imaged at different times post-metamorphosis and scored for the presence or absence of morphological structures to establish a staging scheme. Representative drawings showing each of these stages are shown in Fig. 1c, based on tracings of stained individuals.

### Immunohistochemistry and imaging

Juvenile and late stage *P. lividus* larvae were fixed and stained following protocol in [45] and described in Additional file 1: Methods. Primary and secondary antibodies used are found in Additional file 4: Table S1. Specimens were imaged using a Zeiss LSM 700 confocal microscope, Leica SPEinv inverted confocal microscope using sequential scanning, or Zeiss Lightsheet Z1 microscope.

### Assays of skeletal growth and cell proliferation

To visualize newly deposited CaCO_3_ of the skeleton, live animals were incubated with Calcein (Sigma) as described in [41]. To understand the spatial distribution and quantity of proliferating cells during juvenile sea urchin growth assays of cell proliferation were carried out using the Click-iT® EdU Alexa Flour® 555HCS (Life Technologies) and Click-iT™ EdU Cell Proliferation Kit for Imaging Alexa Flour™ 647 (Thermo Fisher Scientific) following [41]. Cells were quantified for analyzed images using ImageJ and R. Additional details of calcein staining, proliferative staining and imaging can be found in Additional file 1: Methods.

## Supplementary Information

**Additional file 1: Fig. S1.** Confocal images of pre-metamorphosis, late stage *P. lividus* larvae. Shows developing adult body plan within the rudiment stained for Msp130 using monoclonal antibody 6a9 (green), and Sm50 using an anti-Sm50 antibody (magenta). (a) Merged image of Sm50 and Msp130 and nuclear staining (DAPI, blue) showing developing skeletal tissues, within rudiment including spines, tubercles, and the rosette of a primary podium. DAPI (blue) Msp130 strongly labels the bodies and projections of cells tightly associated with the skeleton (skeletogenic cells), and the skeleton. Conversely, staining for Sm50 is stronger in the skeleton itself, and in structures such as the spines and rosette. (b) Interpretive drawing showing morphology corresponding to (a). (c) Magenta channel showing staining for Sm50. Stronger staining of Sm50 is identified at the base of the spines and rosette, where deposition of skeletal tissue is active. In contrast, the larval skeleton is not strongly labeled. (d) Green channel showing staining for Msp130 using monoclonal antibody 6a9. Note the strong staining in the larval skeleton, and the staining of the skeletogenic cells. sc, skeletogenic cells; ls, larval skeleton; pp, primary podium; ros, rosette. Scale bar is 50 μm. **Fig. S2.** Post-metamorphic *P. lividus* juveniles*.* Light microscope images showing post-metamorphic juvenile *P. lividus*. (a) Oral view of stage J4 juvenile. Sphaeridium is well developed and oral surface is dominated by tube feet. Pigmented cells are abundant in the distal disk of the tube feet. (b) Lateral view of J5 animal. Animal is oblong in lateral view, and the aboral surface bears far fewer structures than the oral surface. (c) Oral view of same animal as (b). Note well-developed, circular peristomial margin. Pedicellariae are present on interambulacral plates, and well-developed, glossy sphaeridia are present on ambulacral plates in addition to ambulacral spines. (d) Aboral surface as same animal in (b)-(c). The aboral surface bears few structures, except for the juvenile spines present on genital and ocular plates. Darkened crescent is the gut, visible inside of the animal. sp, secondary podia; sph, sphaeridia; ps, primary interambulacral spine; js, juvenile spine; ped, pedicellariae; as, ambulacral spine. **Fig. S3.** Skeleton and Musculature of *P. lividus* juveniles. Light sheet microscope images showing staining in J1 individual for Myosin Heavy Chain (MHC) using an anti-MHC antibody and Msp130 using monoclonal antibody 6a9. (a) Lateral view showing staining for MHC (Magenta), Msp130 (Green), and nuclei using DAPI (Blue). Msp130 strongly stains the interambulacral primary spines and the juvenile spines, while MHC is localized in the muscular tissue of the primary podia, in longitudinal bands surrounding the tubercles, in bundles within the spines, and in the bases of the pedicellariae valves. (a’) Magenta channel from (a) showing only MHC. (a’’) Magenta and blue channels showing MHC and DAPI. Staining for MHC is strongest in the bases of the pedicellariae valves. (b) Close-up of primary interambulacral spine showing bundles of MHC + cells within the lumen of skeletal spines. (b’) Magenta channel from (b), showing bundles of MHC + cells. This image is the same as Fig. 2b in the main text. (b’’) Bundles of MHC + cells relative to DAPI staining, indicating that each MHC + circle stains multiple cells as opposed to a single cell. (c) Enlargement of pedicellarial staining. The most intense staining for MHC in the animal is the base of the pedicellariae pincers (valves). (d) Close-up from (a) with only magenta and green channels showing longitudinal bands of MHC + cells surrounding the tubercles of primary interambulacral spines and muscles along the primary podia. ps, primary interambulacral spine; js, juvenile spine; pp, primary podia; ped, pedicellariae. Scale bars in (a)-(a’’) are 100 μm, rest are 25 μm. **Fig. S4.** Musculature and morphology of *P. lividus* juveniles. Confocal microscope images showing the distribution of MHC + tissues relative to tissues immunoreactive to an anti-β-tubulin antibody and cell nuclei, marked with DAPI. (a) Image showing a lateral view of MHC immunoreactive cells relative to tissues marked with anti-β-tubulin and DAPI in a J1 individual. MHC + tissues are present throughout the animal, with strong immunoreactivity in the pedicellariae, in longitudinally elongate-cells in the tubercles, and in longitudinal bands alongside the interior of the podia. This image is the same as Fig. 2a in the main text. (b) Oral view showing location of MHC + and β-tubulin + tissues in a J2 *P. lividus*. As in the lateral view, MHC immunoreactive cells are clearly seen around the tubercles and alongside the interior of the podia. In the oral view, MHC + cells are also seen in latitudinal bands around the opening of the mouth in the peristomial membrane. Additionally, muscles involved in the protraction and retraction of the Aristotle’s lantern are visible. (c) Same image as (a), showing only the channel with MHC. Note, a few MHC + cells in the spines. (d) Single-channel image showing MHC channel from (b). Protractor and retractor muscles are visible within the animal, as well as interpyrimidal muscles of the Aristotle’s lantern. (d’) Close-up showing single MHC + cells in the primary spines. Compare with Figure (S3b). (e) Lateral view showing the R-tubulin channel from (a). β-tubulin marks cilia on the outside of the animal, as well as tissues of the nervous system throughout the animal, including around the tubercles. (f) Oral view showing R-tubulin + cells. R- tubulin channel from (b). β-tubulin + cells are located throughout the animal, including in the tips of the podia. Abbreviations are as follows: ps, primary interambulacral spine; pp, primary podia; ped, pedicellariae; tub, tubercle; ipm, interpyrimidal muscle; pr, protractor muscle; ret, retractor muscle. All scale bars are 100 μm, except for (d’), which is 50 μm. **Fig. S5.** Localization of neuronal markers in early juveniles. Confocal microscope images showing staining in J1 individual for synaptotagmin using 1E11 and serotonin using an antiserotonin antibody. (a) Oral view of J1 individual showing immunoreactivity for synaptotagmin (green), and serotonin (magenta). Nuclei are stained with DAPI (blue). Synaptotagmin is localized within the interior of the primary podia, and in bands surrounding the tubercles and bases of the spines. Synaptotagmin and serotonin co-localize in single cells arranged proximodistally along the lumen of the primary interambulacral spines. This is the same as Fig. 2d in the main text. (b) Close-up of (a). (c) Aboral view of same individual as (a). (d) Green channel from (a) showing synaptotagmin. (e) Green channel from (b) showing synaptotagmin. Note the synaptotagmin + cells lining the lumen of the primary interambulacral spines. (f) Green channel from c showing synaptotagmin. (g) Magenta channel from a showing serotonin. (h) Magenta channel from (b) showing serotonin. Note the synaptotagmin + cells lining the lumen of the primary interambulacral spines, which we interpret to be serotonergic neurons. (i) Magenta channel from (c) showing Serotonin. (j) Green and blue channels from (a) showing synaptotagmin and DAPI. (k) Green and blue channels from (b) showing synaptotagmin and DAPI. (l) Green and blue channels from (c) showing synaptotagmin and DAPI. All scale bars in (c), (f), (i), (l) are 50 μm, rest are 100 m. **Fig. S6**. Neuronal and skeletal markers in early juveniles. Confocal images showing localization of ELAV and Msp130 in J2 individual. (a) Staining for ELAV using an anti-ELAV antibody (Magenta), Msp130 using monoclonal antibody 6a9 (Green), and DAPI (blue). Msp130 is strongly localized in the primary and juvenile spines, and the rosettes while ELAV is localized in cells in the primary and secondary podia. (b) Close-up of same individual as (a) showing ELAV + cells, interpreted to be neurons, in the secondary podia. In the interambulacral primary spines, Msp130 is localized in the cell bodies of cells that we interpret to be skeletogenic cells. This image is the same as Fig. 2c in the main text. (c) Magenta and green channels of (a), showing staining of ELAV and Msp130. (d) Magenta and green channels of (b). Importantly Msp130 and ELAV are differentially localized in the secondary podia, with ELAV marking neurons, and Msp130 staining the growing rosette. (e) Magenta and blue channels of (a), showing ELAV and DAPI. (f) Magenta and blue channels of (b), showing ELAV and DAPI. Cell nuclei are marked by DAPI, but ELAV stains the surrounding cell body. (g) Green and blue channels of a, showing MSP130 and DAPI. (h) Green and blue channels of (b), showing Msp130 and DAPI. As with ELAV, Msp130 stains the cell body, while nuclei of the same cells are marked with DAPI. n, neuron; ros, rosette; sc, skeletogenic cell. Scale bars in (a), (c), (e), (g) are 200 μm, scale bars in (b), (d), (f), (h) are 100 μm. **Fig. S7.** Specific and pan-neuronal markers. Confocal images showing localization of ELAV and Synaptotagmin in J1 individual. (a) Staining for synaptotagmin (green). Notice rings of nerves surrounding tubercles and in bundles of neurons in the primary spines. (b) Stainingfor ELAV. Notice bundles of neurons in primary spines. (c) DAPI staining showing distribution of cell nuclei. (d) Merge showing co-localization of Synaptotagmin and ELAV. Of importance is the fact that co-localization is most strong in the bundles of neurons at the base of the primary spines. Scale bars in (a)-(c) are 200 μm, (d) is 50 μm. **Fig. S8** Localization of acetylated tubulin. Confocal images showing staining for acetylated tubulin and DAPI in J1 animals. (a) Merge showing staining for acetylated tubulin (green) using an anti-acetylated tubulin antibody and for cell nuclei using DAPI (blue) on the oral surface. Cilia cover the entire surface of the animal and the lateral edges of the spines, as shown in Gosselin and Jangoux [1]. Acetylated tubulin is also found in the interior of the primary and secondary podia, which we interpret to be tissue of the nervous system in agreement with a recent study [2]. (b) Staining for acetylated tubulin and cell nuclei on the aboral surface of a different J1 individual. Cilia are found across the surface of the animal, and show a high density on pedicellariae. The individual from (a) is shown in the bottom of the image. (c) Green channel from a showing only staining for acetylated tubulin. Note the cilia lining the margin of the discs of the primary podia. (d) Green channel from (b) showing staining of acetylated tubulin. pp, primary podia; sp, secondary podia; js, juvenile spine; ps, primary interambulacral spine; ped, pedicellariae. Scale bar in all images is 200 μm. **Fig. S9** Localization and co-localization of skeletal proteins in young juveniles. Localization of the skeletogenic proteins Msp130 (green) and Sm50 (purple) in a J1 *P. lividus* juvenile. (a)-(c)- show localization of Msp130 and Sm50 relative to DAPI (blue), which stains for nuclei. (d)-(f) show both distinct localization, and co-localization of Msp130 and Sm50 in skeletal tissues. (g)-(i) show localization of Sm50, staining is strongest around the margins of plates, in median portions of spines, and in milled-rings of spines. (j)-(l) Staining of Msp130 in skeletal tissues. Staining is strongest in the tips of spines. pp, primary podia; sp, secondary podia, ps, primary interambulacral spine; ped, pedicellariae. These images are the same as those shown in Fig. 2il in the main text. Scale bars 200 μm. **Fig. S10.** Calcein localization relative to skeletal proteins. Confocal microscopy showing staining of Sm50 using an anti-Sm50 antibody (magenta), and Msp130 using monoclonal antibody 6a9 (green), as well as the incorporation of fluorescent calcein (yellow) into the growing J3 sea urchin test. (a) Aboral surface showing incorporation of calcein into the margins of accreting genital, anal, ocular and interambulacral plates. Msp130 and Sm50 are also shown, and reveal distinct patterns of localization and co-localization. (b) Oral surface of same individual shown in (a). Calcein is shown incorporated into margins of accreting ambulacral and interambulacral plates, elongating sphaeridia, growing hemipyramids, and rosettes. There is co-localization of Sm50 and Msp130 in sites of calcein incorporation, implicating Sm50 and Msp130 in active skeletogenesis. (c) Same as (a), with calcein removed. Co-localization of Msp130 and Sm50 is indicated by greyish-white color. This is evident at the margins of genital and interambulacral plates, as well as in the tubercles. Compared with (a) and (e), it is evident that strong co-localization of these skeletogenic genes occurs in sites of accretion as identified using calcein. (d) Same as (b), with calcein removed. Co-localization of Msp130 and Sm50 is evident in the margins of accreting peristomial, ambulacral, and interambulacral plates, as well as in the elongating sphaeridia and rosettes of secondary podia. Compared with (a), and (f), this co-localization is evident in sites of calcein incorporation. (e) Yellow channel from (a), showing sites of calcein incorporation in genital, interambulacral and anal plate margins. Compare with co-localization of skeletogenic proteins in (c). (f) Yellow channel from (b), showing sites of calcein incorporation in margins of accreting peristomial, ambulacral, and interambulacral plates, and growing hemipyramids, rosettes and sphaeridia. Compare with sites of skeletogenic protein localization in (d), (h), and (j). (g) Purple channel from (a), showing localization of Sm50 protein. Immunoreactivity is stronger in the bases of spines and tubercles, and in the margins of genital, anal and interambulacral plates. (h) Purple channel from (b), showing localization of SM50. Immunoreactivity is stronger in margins of peristomial plates, elongating sphaeridia, and rosettes of secondary podia. (i) Green channel from a showing localization of Msp130 proteins using 6a9. Immunoreactivity is stronger in the medial and distal portions of interambulacral spines, and in the distal portions of juvenile spines and pedicellariae. (j) Green channel from (b) showing localization of Msp130 protein using 6a9. Localization is strongest in medial and distal portions of interambulacral spines, in the rosettes, in elongating sphaeridia and in the margins of peristomial plates. ps primary spine; js, juvenile spine; ped, pedicellariae; sph, sphaeridia, pp, primary podia; sp, secondary podia; hp, hemipyramid. These images are the same as those shown in Fig. 3a-b of the main text. Scale bar in all images is 200 μm. **Fig. S11.** Skeletal growth over time. Localization of Msp130 (green), Sm50 (purple), and incorporation of calcein (yellow) into growing skeleton in J3 individuals at 0 (a-a’’’), 24 (b-b’’’), and 48 (c–c’’’) hours chase. Description of staining given in the main text. a’, b’, c’ and a’’’, b’’’, and c’’’ are zoomed in images of the above showing incorporation of calcein. Orange bars indicate gap between calcein-marked plated due to subsequent accretion. ps primary spine; js, juvenile spine; op, ocular plate, gp, genital plate; ap, anal plate; sph, sphaeridia. Images are the same as individuals in Figs. 3a,b,d,e in the main text. Scale bars in a-c and a’’-c’’ 200 μm, a’, b’’’, c’, c’’ 25 μm, b’ and a’’’ 50 μm. **Fig. S12.** Calcein and Sm50. Confocal microscopy showing staining for Sm50 using an anti-Sm50 antibody (magenta), and the incorporation of fluorescent calcein (yellow) into the J3 sea urchin test at 0, 24, and 48 h chase. (a) Aboral surface from 0 h chase animal showing incorporation of calcein) relative to localization of Sm50. Image is the same as Fig. 3a in the main text, with green channel (MSP130) removed. Sm50 is localized throughout all visible skeletal structures, including localization in areas of calcein incorporation such as the margins of plates, shown in whitish-grey. (a’) Yellow channel from (a), showing sites of calcein incorporation in aboral surface of 0 h chase animal. (a’’) Oral surface from 0 h chase animal, showing incorporation of calcein into skeleton relative to localization of Sm50. Sm50 stains the entire skeleton except for the teeth, and is localized in sites of calcein incorporation in the sphaeridia, rosettes, and margins of peristomial, ambulacral, and interambulacral plates. Image is the same as Fig. 3b in the main text, with green channel (Msp130) removed. (a’’’) Yellow channel from (a’’) showing incorporation into oral surface of 0 h chase animal. (b) Aboral surface of 24 h chase animal showing incorporation of calcein relative to staining for Sm50. Additional skeletogenesis at the margin of plates has taken place in the 24 h since calcein incubation, as evident in the gaps between calcein-incorporated skeleton in adjacent plates. Sm50 is still strongly localized in some areas of calcein incorporation, such as the tubercles and margins of plates. Image is the same as Fig. 3d in the main text, with green channel (Msp130) removed. (b’) Yellow channel from (b), showing incorporation of calcein into aboral surface of 24 h chase animal. (b’’) Incorporation of calcein and localization of Sm50 protein in oral surface of 24 h chase animal. Sm50 is localized where calcein was incorporated into the skeleton in the ambulacral, peristomial and interambulacral plates, but strong Sm50 localization is also evident in skeleton that has been deposited subsequent to incubation with calcein. This is most evident in the margins of the peristomial plates, in the elongating sphaeridia, and in the rosettes of secondary podia. (b’’’) Yellow channel showing incorporation of calcein in the skeleton of the oral surface of a 24 h chase animal. (c) Incorporation of calcein and localization of Sm50 in the skeleton of the aboral surface of a 48 h chase animal. Gaps between sites of calcein incorporation in adjacent plates (of maximum size of 7.5 μm) are clearly visible on the aboral surface. Additionally, strong Sm50 localization in these gaps, and in other structures such as the tubercles, bases of primary spines, and margins of plates are indicative of further biomineralization involving Sm50. (c’) Yellow channel from c showing incorporation of calcein into aboral skeleton of 48 h chase animal. Image is the same as zoom of Fig. 3e in the main text. (c’’) Oral surface of 48 h chase animal showing incorporation of calcein and localization of Sm50. Substantial growth has taken place in the 48 h since the incorporation of calcein, which is evident in the strong localization of Sm50 in the sphaeridia, in themargins of the peristomial plates, and in the rosettes of secondary podia. Additionally, gaps between areas of calcein incorporation are present in ambulacral and interambulacral plates. (c’’’) Yellow channel of (c’’) showing incorporation of calcein into oral surface of 48 h chase animal. iamb, interambulacral plate; op, ocular plate; sph, sphaeridia; pp, primary podia; sp, secondary podia. Scale bar in all images is 200 μm. **Fig. S13.** Skeletal growth in older (J6) juvenile. Localization of SM50 (purple), MSP130 (green) and incorporation of calcein (yellow) into J6 animal at 0 h chase. (a) shows staining of MSP130, SM50, and incorporation of calcein into oral surface. This is the same image as Fig. 3c in the main text. (b) is yellow channel from (a) showing calcein in the Aristotle’s lantern, rosettes, and spines. (c) is the aboral surface of the same J6 animal from (a). (d) shows the yellow channel with calcein from (c). (e–f) are close-ups of the aboral surface from (c) showing incorporation of calcein into growing spines and tubercles relative to MSP130 and SM50 (e) and only SM50 (f). (e) is the same as Fig. 3f from the main text. ps primary spine; sp, secondary podia; sph, sphaeridia; ped, pedicellariae; aps, ambulacral primary spine. Scale bars in a-d 200 μm and e–f 50 μm. **Fig S14.** Images showing different stages of the strategy used to quantify EdU + nuclei relative to all nuclei. (a) and (b) Show raw images for EdU + (a) and DAPI + cells (b). (c-d) Binary images for quantification. (e–f) Five regions of interest of binary cells for quantification. See expanded details in Supplemental Methods. Image is the same as Fig. 4b from the main text. **Fig. S15.** Cell proliferation pulse-chase in early juvenile stages. Confocal microscope images showing the incorporation of EdU into the oral surface of growing J2 sea urchins, as well as staining for Sm30 using an anti-SM30 antibody. (a) Staining for EdU (magenta), Sm30 (gray), and cell nuclei using DAPI (blue) in the oral surface of a 0-h chase individual. Most proliferation takes place in the secondary podia and growing peristomial podia. Additionally, immediately adoral to the primary podia, there is a zone of cell proliferation corresponding to the zone where new plates are added. Of interest, there is no proliferation in the primary podia themselves, which form in the rudiment and atrophy within a few weeks following metamorphosis [1]. (a-a- ‘’) are the same image as Fig. 4b from the main text. (a’) ray channel from a showing localization of Sm30. Protein is localized in all skeletal tissue except for the teeth and is most strongly localized in the primary interambulacral spines. (a’’) Localization of Sm30 relative to proliferating cells marked by EdU. There is very little proliferation in the primary spines and in the peristomial test plates. (b) Staining for EdU, Sm30, and cell nuclei using DAPI in a 24 h chase individual. As in the 0 h chase individuals, most proliferating cells are located in the secondary and peristomial podia, and the proliferative zone adoral to the primary podia. (b’) Gray channel from (b), showing localization of Sm30. (b’’) Localization of Sm30 relative to proliferative cells marked by EdU. (c) Staining for EdU, Sm30, and cell nuclei using DAPI in a 48 h chase individual. Localization of EdU + cells does not differ substantially from 0 and 24 h chase individuals. Presence of cell doublets indicates that rates of cell division are relatively slow, and that not much cell division has taken place in the 48 h since incubation with EdU. (c’) Gray channel showing localization of Sm30. (c’’) Location of EdU + cells relative to skeletal tissues identified using Sm30. ps, primary interambulacral spine; pp, primary podia; sp, secondary podia. Scale bar in all images is 200 μm. **Fig. S16.** Cell proliferation in J5 Juvenile. Incorporation of EdU into a 0 h chase J5 individual after 63 h of incubation with EdU, and staining for S530 using an anti-SM50 antibody. (a) Aboral surface of 0 h chase animal showing localization of Sm50 relative to proliferating cells marked by EdU (magenta-white). There is relatively little cell proliferation present in extraxial tissues on the aboral surface such as the genital and periproctal (anal) plates. Most cell proliferation seen from this view is associated with novel structures, such as newly added secondary podia and ambulacral spines. (b) Proliferating cells marked by EdU and localization of Sm50. Proliferation is extensive on the oral surface in axial tissues, especially when compared to the extraxial tissues of the aboral surface. High degrees of cell proliferation are found in the disks of the secondary podia, as well as in the peristomial podia and sphaeridia. Most proliferating cells on spines are located more proximally towards the base of the spine. (c) Magenta channel from (a), showing location of proliferating cells on aboral surface. (d) Magenta channel from (b), showing location of proliferating cells on oral surface. (e) Close-up view of cell proliferation associated with growth of a newly added ambulacral primary spine. Most proliferating cells are localized towards the base of the spine and the tubercle. Strong staining of Sm50 is also located towards the base of the spine, though we interpret most distal growth takes place via skeletogenesis. (e) is the same as Fig. 4i in the main text. (f) Cell proliferation associated with a growing primary spine and secondary podia. As in (e), most proliferating cells are located nearer to the base of the spine. High degrees of cell proliferation are associated with the growth of the secondary podia, as is also seen in J2 individuals in Fig. 4b. (f) is the same as Fig. 4j in the main text. (g) On the oral surface, high degrees of cell proliferation are associated with the tube feet. In particular, the margins of the tube feet disk show high degrees of cell proliferation, potentially associated with sensory motor neurons. ps, primary interambulacral spine; aps, ambulacral primary spine; sp, secondary podia; ped, pedicellariae; m, mouth. Scale bars in (a)-(d) are 200 μm, scale bars in (e)-(g) are 100 μm. **Fig. S17.** Cell proliferation in a J6 Juvenile. Incorporation of EdU into a 48 h chase growing J6 sea urchin after 63 h of incubation with EdU. (a) EdU stained nuclei (magenta) relative to all cell nuclei marked with DAPI (gray) on the aboral surface. Note the low abundance of proliferating cells on extraxial tissue (genital and anal plates). Most cell proliferation in this view is associated with the addition of new structures, such as ambulacral and interambulacral primary spines. (b) Cell proliferation marked using EdU on the oral surface of a J6 animal. High degrees of cell proliferation are associated with newly added pedicellariae. Proliferating cells are also found in the circular margins of the disks of the secondary podia. (c) Magenta channel of (a), showing location of cells marked with EdU. (d) Magenta channel of (b), showing EdU marked cells on oral surface. (e) Close-up of newly added ambulacral and interambulacral spines, secondary podia, and associated cell proliferation. (f) Close-up of proliferating cells in newly added secondary podia and spines. (g) Close-up of ambulacral pedicellariae and associated cell proliferation. ps, primary interambulacral spine; aps, ambulacral primary spine; sp, secondary podia; ped, pedicellariae; m, mouth. Scale bars in (a)-(d) are 200 μm, scale bars in (e)-(g) are 100 μm.

**Additional file 2.** R script to repeat statistical analyses of EdU labeled nuclei.

**Additional file 3: Table S2.** Quantification of EdU + nuclei relative to all nuclei labeled with DAPI.

**Additional file 4: Table S1.** Antibodies and dilutions used in this study.

## Data Availability

All supporting data, and code, is available as part of the in the supplemental material.
